# Trial protocol: DOLFIN trial: Developmental Outcomes of Long-term Feed Supplementation in Neonates—A UK multicentre, blinded, stratified, randomised controlled trial

**DOI:** 10.1186/s13063-025-09253-3

**Published:** 2025-12-29

**Authors:** Morag J. Andrew, Nicholas Embleton, Pollyanna Hardy, Samantha Johnson, Edmund Juszczak, Heather Ledbury, Claire Pearson, Oliver Rivero-Arias, Adriana Amorim Francisco, Janet Berrington, Penny Bradley, Peter J. Bradley, Christina Cole, Kate Court, Madeleine Hurd, Andrew King, Louise Linsell, David Murray, Heather M. O’Connor, Charles C. Roehr, Victoria Stalker, Kayleigh Stanbury, Usharani Devi Wahengbam, Richard Welsh, Joy Wiles, Jeremy R. Parr

**Affiliations:** 1 Population Health Sciences Institute, Newcastle University, Newcastle upon Tyne, Tyne and Wear UK; 2 Newcastle upon Tyne Hospitals NHS Foundation Trust, Royal Victoria Infirmary, Queen Victoria Road, Newcastle upon Tyne, UK; 3 National Perinatal Epidemiology Unit Clinical Trials Unit, Nuffield Department of Population Health, University of Oxford, Old Road Campus, Oxford, UK; 4 Department of Population Health Sciences, University of Leicester, Leicester, UK; 5 Nottingham Clinical Trials Unit, School of Medicine, University of Nottingham, Nottingham, UK; 6 Portsmouth Hospitals University National Health Service Trust, Portsmouth, UK; 7 Translational and Clinical Research Institute, Faculty of Medical Sciences, Newcastle University, Newcastle upon Tyne, UK; 8 Bliss, 1st Floor North, 10-18 Union Street, London, SE1 1SZ UK

**Keywords:** Preterm, Hypoxic ischaemic encephalopathy, Neurodevelopment, Cognition, Brain, Nutrition, Nutrient, Docosahexaenoic acid, Choline

## Abstract

**Background:**

Infants born extremely preterm (EP; < 28 weeks of gestation) or term born infants with hypoxic-ischemic encephalopathy (HIE) have increased risk of long-term cognitive and learning deficits. Early supplementation with long chain polyunsaturated fatty acids (LCPUFAs) docosahexaenoic acid (DHA), eicosapentaenoic acid (EPA), and arachidonic acid (ARA), choline, uridine-5′-monophosphate (UMP), and cytidine-5′-monophosphate (CMP), zinc, iodine, and vitamin B12 may improve cognitive and language outcomes in these populations.

**Methods:**

This multicentre, blinded, stratified, randomised controlled trial, including an economic evaluation, will investigate the impact of nutritional supplementation on cognitive development in infants born EP or term born infants with HIE. The planned sample size is 1010 (538 EP, and 472 HIE) infants from up to 40 National Health Service neonatal units in the UK. The trial patient populations are infants born EP (preterm stratum) and term infants (born at or more than 35 weeks of gestation) with HIE who received therapeutic hypothermia (HIE stratum). Patient strata were chosen to include infants at high risk of adverse neurodevelopmental outcomes by virtue of EP birth, or HIE requiring therapeutic hypothermia. Infants are randomly assigned, in a 1:1 allocation ratio, to receive either the active supplement or a matched control, in addition to standard care. Families, clinical teams, investigators, and Clinical Trials Unit staff are blinded to allocation. Only the Senior Trials Programmer and Trial Statisticians have access to allocation information. The active supplement is a nutrient powder formulated to be mixed with breast milk, infant formula, or food, containing LCPUFAs (including DHA, EPA, and ARA), choline, UMP, CMP, zinc, iodine, and vitamin B12. Supplementation commences once infants achieve full milk feeds and continues until 12 months post-estimated date of delivery (EDD), with a daily dosage of 1 g per kilogram of body weight. The primary outcome is the Parent Report of Children’s Abilities-Revised non-verbal cognitive scale at 24 months post-EDD. EP and HIE patient population comparisons have been appropriately powered and will be analysed separately.

**Discussion:**

Findings from the DOLFIN trial will inform international neonatal and infant nutritional and feeding policy and practice. Learnings from the trial will inform the design and delivery of future neonatal nutritional intervention trials.

**Trial registration:**

ISRCTN62323236. Registered 16 May 2022, https://www.isrctn.com/ISRCTN62323236.

## Administrative information

Note: the numbers in curly brackets in this protocol refer to SPIRIT checklist item numbers. The order of the items has been modified to group similar items (see http://www.equator-network.org/reporting-guidelines/spirit-2013-statement-defining-standard-protocol-items-for-clinical-trials/).

**Table:** 

Title {1}	**DOLFIN Trial**: **D**evelopmental **O**utcomes of **L**ong-term **F**eed Supplementation **i**n **N**eonates. A multicentre, blinded, stratified, randomised controlled trial.
Trial registration {2a and 2b}.	ISRCTN: 62,323,236
Protocol version {3}	V6.0 10/10/2024
Funding {4}	NIHR HTA programme—NIHR130925
Author details {5a}	Morag J Andrew, MBChB, DPhil, BSc Med Sci (Hons)—Honorary Clinical Senior Lecturer and Consultant Paediatrician; Newcastle University and Newcastle Upon Tyne Hospitals NHS Foundation Trust. Nicholas Embleton, MBBS (Hons), MD—Honorary Clinical Professor in Neonatal Medicine and Consultant Paediatrician; Newcastle University and Newcastle Upon Tyne Hospitals NHS Foundation Trust. Pollyanna Hardy, BSc, MSc—Clinical Trialist and Director of the National Perinatal Epidemiology Unit Clinical Trials Unit (NPEU CTU); University of Oxford. Samantha Johnson, PhD—Professor of Child Development—Department of Population Health Sciences; University of Leicester. Edmund Juszczak, MSc—Professor of Clinical Trials & Statistics in Medicine—School of Medicine & Health Sciences; University of Nottingham. Heather Ledbury—Population Health Sciences Institute; Newcastle University. Claire Pearson, BSc—Portsmouth Hospitals University NHS Trust, Portsmouth, UK. Oliver Rivero-Arias, DPhil—Associate Professor of Health Economics and Lead Health Economist—National Perinatal Epidemiology Unit Clinical Trials Unit (NPEU CTU); University of Oxford. Adriana Amorim Francisco, PhD—Research Nurse—National Perinatal Epidemiology Unit Clinical Trials Unit (NPEU CTU); University of Oxford. Janet Berrington, BMBS, MD—Honorary Professor—Translational and Clinical Research Institute, Faculty of Medical Sciences; Newcastle University and Consultant Neonatal Paediatrician; Newcastle University and Newcastle Upon Tyne Hospitals NHS Foundation Trust. Penny Bradley, MPharm—Senior Lead Clinical Trials Pharmacist—Pharmacy Dept, The Newcastle-upon-Tyne Hospitals NHS Foundation Trust Peter J Bradley—Director of Services; Bliss. Christina Cole, BSc, MSc—Senior Trials Manager—National Perinatal Epidemiology Unit Clinical Trials Unit (NPEU CTU); University of Oxford. Kate Court, PhD—Head of Middleware & Data Science; Newcastle University. Madeleine Hurd, BSc (Hons)—Data Manager—National Perinatal Epidemiology Unit Clinical Trials Unit (NPEU CTU); University of Oxford. Andrew King, BA—Head of Trials Programming—National Perinatal Epidemiology Unit Clinical Trials Unit (NPEU CTU); University of Oxford. Louise Linsell, BSc (Hons), MSc, DPhil,—Associate Professor—National Perinatal Epidemiology Unit Clinical Trials Unit (NPEU CTU); University of Oxford. David Murray, BSc—Senior Trials Programmer—National Perinatal Epidemiology Unit Clinical Trials Unit (NPEU CTU); University of Oxford. Heather M O’Connor, BSc (Hons), MSc—Senior Statistician—National Perinatal Epidemiology Unit Clinical Trials Unit (NPEU CTU); University of Oxford. Charles C Roehr, BSc, MD, PhD—Professor of Neonatal and Perinatal Medicine—Bristol Medical School (THS); University of Bristol. Clinical Advisor—National Perinatal Epidemiology Unit Clinical Trials Unit (NPEU CTU); University of Oxford. Victoria Stalker—Project Manager—Department of Psychiatry, Medical Science Division; University of Oxford. Kayleigh Stanbury, BSc (Hons)—Head of Operations—National Perinatal Epidemiology Unit Clinical Trials Unit (NPEU CTU); University of Oxford. Usharani Devi Wahengbam, BSc, MSc—Trial Manager—National Perinatal Epidemiology Unit Clinical Trials Unit (NPEU CTU); University of Oxford. Richard Welsh, BEng (Hons)—Senior Software Developer—National Perinatal Epidemiology Unit Clinical Trials Unit (NPEU CTU); University of Oxford. Joy Wiles, BSc (Hons)—Quality Assurance Manager—National Perinatal Epidemiology Unit Clinical Trials Unit (NPEU CTU); University of Oxford. Jeremy R Parr, MB ChB, MD—Professor of Paediatric Neurodisability—Population Health Sciences Institute, Newcastle University; Newcastle Upon Tyne Hospitals NHS Foundation Trust.
Name and contact information for the trial sponsor {5b}	The Newcastle upon Tyne Hospitals NHS Foundation Trust Newcastle Joint Research Office Level 1, Regent Point Regent Farm Road Gosforth Newcastle upon Tyne NE3 3HD Email: tnu-tr.sponsormanagement@nhs.net Telephone: 01912824454
Role of sponsor {5c}	The sponsor will be responsible for overseeing the clinical trial to ensure it meets regulatory and ethical standards. They will provide indemnity arrangements, ensure the trial is conducted in compliance with Good Clinical Practice and all applicable regulations. The Sponsor does not participate in the analysis, interpretation of preparation of results, but will review publications prior to dissemination.

## Introduction

### Background and rationale {6a}

Children born extremely preterm (EP; < 28 weeks of gestation) have substantial cognitive deficits that are present in infancy and persist throughout childhood and adolescence, with deficits in intelligence quotient (IQ) of a similar magnitude in adulthood as in childhood [1, 2]. Up to 40% of EP children born at less than 26 weeks of gestation have moderate/severe learning disability (sometimes referred to as intellectual disability) and 2 in 3 have special education needs (SEN) by the end of primary education [3]. EP birth may also be associated with reduced quality of life and well-being in adulthood [4, 5], alongside reduced wealth, occupational status and economic potential [6].

Hypoxic-ischemic encephalopathy (HIE) is a clinical diagnosis of babies born at or near term. HIE often results in lifelong disability even in the absence of radiological abnormalities. Cognitive deficits, particularly in attention and executive functions, are identified in children following moderate-severe HIE [7–9]. HIE-affected children without cerebral palsy are also at increased risk of cognitive impairments [10]. The impact of these deficits on school attainment and social functioning are significant [10].

Rapid brain growth and development during the third trimester of pregnancy and the first 2 years of life relies upon adequate nutritional intake. Dietary supply of long chain polyunsaturated fatty acids (LCPUFAs), choline, and uridine-5′-monophosphate (UMP), are particularly important for healthy brain development, being key to numerous brain processes, including phospholipid production for new neural membranes, neurogenesis and synaptogenesis [11]. Provision of these nutrients in combination has a synergistic effect [12, 13]. Micronutrients, including zinc, the B vitamins, and iodine, also have important central nervous system roles [14].

Following brain injury, plasticity may contribute to functional recovery, supported by the synaptic elements, extracellular matrix, and guidance molecules of the cortical subplate [15]. There is growing interest in the potential for targeted nutritional interventions to ameliorate the neurodevelopmental impacts of early brain insult by optimising the developing brain’s inherent plasticity and repair mechanisms. Provision of these nutrients in combination resulted in reduced lesion size in a mouse model of neonatal hypoxic ischaemic brain injury [16].

Population research indicates that docosahexaenoic acid (DHA) and eicosapentaenoic acid (EPA) intakes are below recommended daily amounts in women of childbearing age [17]. During pregnancy, the majority of transplacental DHA transfer occurs during the third trimester and so infants born EP miss out on this. Following EP birth, multiple additional challenges contribute to a growing DHA ‘gap’, including prolonged use of parenteral nutrition, and interruptions to enteral feeding due to intercurrent illness [18].

In this context, there has been substantial interest in whether early-life DHA supplementation improves neurodevelopmental outcomes in infants, including those at risk of neurodevelopmental impairment [19]. Historically, trials of infant DHA supplementation have been unable to demonstrate definitive neurodevelopmental benefits, in part due to heterogeneity in trial design including DHA dosing and duration of supplementation [20]. Subsequently, high dose DHA supplementation of infants born before 29 weeks of gestation who participated in the N-3 Fatty Acids for Improvement in Respiratory Outcomes (N3RO) randomised controlled trial assessing the effect of DHA supplementation on bronchopulmonary dysplasia (BPD) had higher Full Scale IQ scores at 5 years of age compared with controls [21]. Concerns about possible increased rates of BPD in supplemented infants in the N3RO and Maternal Omega-3 Supplementation to Reduce Bronchopulmonary Dysplasia in EP Infants (MOBYDIck) trial [22] have been resolved through individual patient data meta-analysis which confirms high dose DHA was not associated with increased BPD risk in EP infants [23].

Given the positive synergistic effects of LCPUFAs, choline, and nucleotides and the relevance of micronutrients for neurodevelopment, two exploratory randomised controlled trials (RCTs) were conducted to investigate whether intervention with this specific nutrient combination was feasible in infants at risk of neurological impairment: The Dolphin Neonatal RCT and the Dolphin Infant RCT [24, 25]. Both used a nutritional supplement (a nutrient blend containing long chain polyunsaturated fatty acids—DHA, EPA and arachidonic acid (ARA), choline, UMP, and cytidine-5′-monophosphate (CMP), zinc, iodine, and vitamin B12), given for 24 months in newborns and infants at risk of neurological impairment [26]. The primary outcome was the Bayley Scales of Infant and Toddler Development III [27] Cognitive Scale score, administered following supplementation for 24 months. In the neonatal RCT, the treatment group (preterm infants (< 31 weeks of gestation) and term infants with HIE, *n* = 62) had higher mean cognitive scale scores (mean difference, 9·0; 95% confidence interval (CI), −0·2 to 18·2) and language scale scores (mean difference, 8·6; 95% CI, −1·1 to 18·2) compared to the control group. Parent reports of neurodevelopmental outcomes showed similar results [23]. The parallel infant RCT (infants aged 1 to 18 months with suspected or confirmed cerebral palsy; *n* = 40) reported similar findings [25]. The subsequent pre-school follow up study of Dolphin Neonatal and Infant RCT participants at age 4–6 years showed treatment group advantage (increase of 8.9 IQ points; 95% CI, −4.4 to 22.2) in cognitive development (assessed by the Kaufman Assessment Battery for Children II) [28] compared to controls (Andrew MJ, in preparation). Treatment group advantage in visual attention was observed in Dolphin Neonatal RCT participants [29]. In combination, these studies suggest a potential treatment effect from the nutritional supplement; however, these small studies were not sufficiently powered. We therefore designed the DOLFIN study to definitively investigate the clinical and cost-effectiveness of the nutritional supplement for breastfed and/or infant formula fed infants at high risk of adverse neurodevelopmental outcomes, provided for at least the first year of life.

## Objectives {7}

### Primary objective

The primary objective of the DOLFIN trial is to compare the cognitive development of infants randomised to receive nutritional supplementation with a nutrient blend containing LCPUFA, choline, UMP, CMP, zinc, iodine, and vitamin B12 compared to those randomised to receive matched control supplement, at 24 months post-estimated date of delivery (EDD).

### Secondary objectives

The secondary objectives of the DOLFIN trial are to as follows:

- To compare the effects of nutritional supplementation with a nutrient blend containing LCPUFAs, choline, UMP, CMP, zinc, iodine, and vitamin B12 with a matched control supplement on secondary neurodevelopmental outcomes (PARCA-R language development scale, Strengths and Difficulties Questionnaire (SDQ), Ages and Stages Questionnaire (ASQ-3)), at 24 months post-EDD.
- To investigate the effect of nutritional supplementation with a nutrient blend containing LCPUFAs, choline, UMP, CMP, zinc, iodine, and vitamin B12 on the following:
  1. Infant growth outcomes to 24 months post-EDD (weight standard deviation (SD) score, body mass index (BMI) ≥85th percentile, head circumference SD score);
  2. Clinical outcomes up to discharge (microbiologically confirmed late-onset invasive infection, necrotising enterocolitis requiring surgery, retinopathy of prematurity treated medically or surgically in preterm infants only, chronic lung disease in preterm infants only);
  3. Safety (serious adverse events), infant tolerability (Infant Gastrointestinal Symptom Questionnaire (IGSQ-13)), adherence and parental acceptability to 12 months post-EDD;
  4. Maternal health-related quality of life up to 24 months post-EDD (EuroQol EQ-5D-5L);
  5. Clinical diagnoses up to 24 months post-EDD (fit or seizure within the last 12 months, presence of a feeding tube of any type, receiving supplemental oxygen or any respiratory support, confirmed or suspected diagnosis of cerebral palsy, hydrocephalus treated with a third ventriculostomy or ventriculo-peritoneal shunt);
  6. Cost-effectiveness up to 24 months post-EDD; and modelled up to 18 years post-EDD (Bespoke Health Economic questionnaire).

**Fig. 1:**
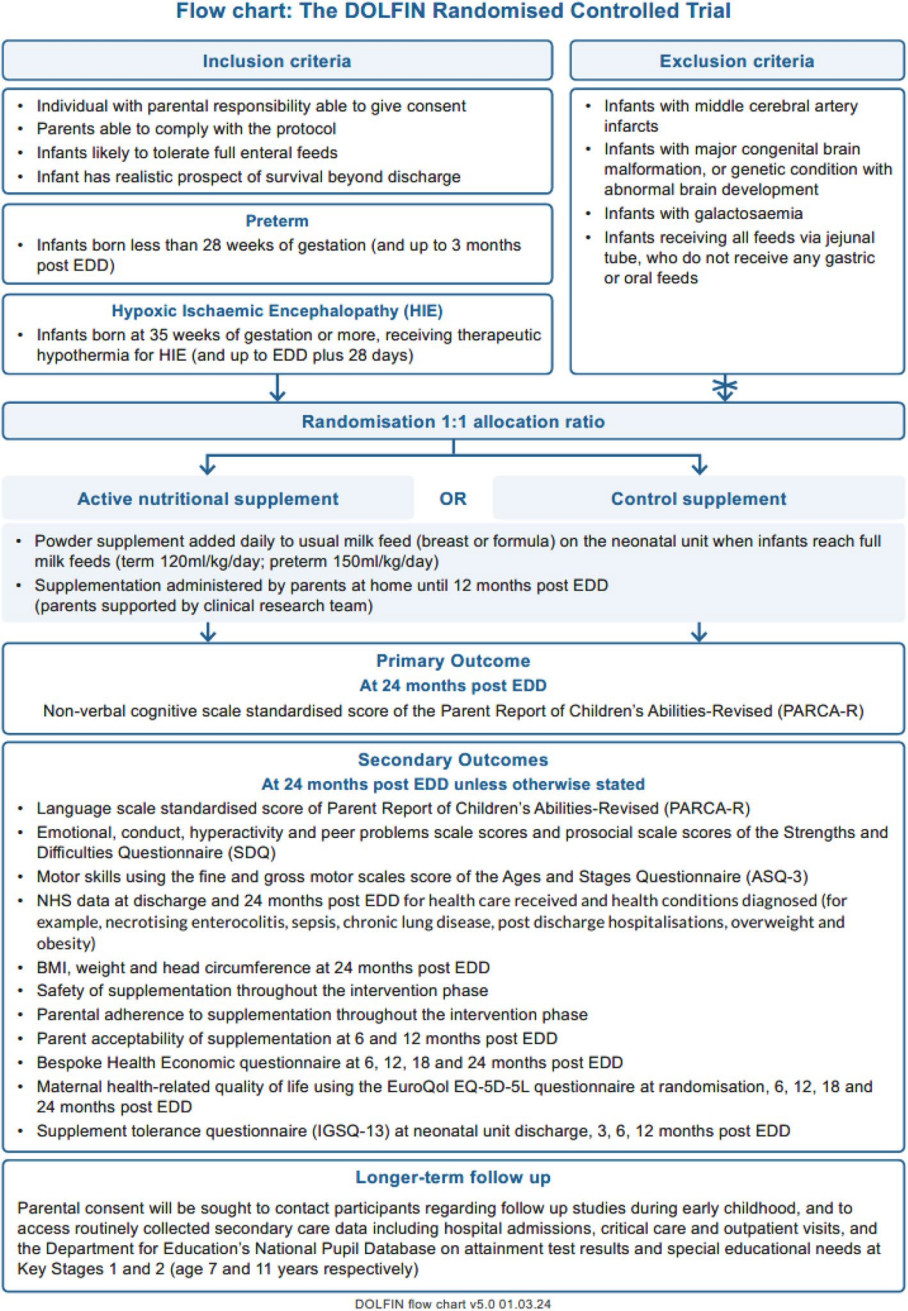
Trial procedure flow chart and list of outcomes at 24-months post-EDD (The two infant populations are treated separately)

For a full list of trial outcomes see Fig. 1.

## Trial design {8}

The DOLFIN trial is a multicentre, blinded, stratified, superiority randomised controlled trial (with internal pilot and an economic evaluation), using an allocation ratio of 1:1. Patient populations (strata) were chosen to include infants at high risk of adverse neurodevelopmental outcomes by virtue of EP birth, or HIE requiring therapeutic hypothermia and are defined as follows:

1. Preterm stratum: infants born < 28 weeks of gestation (and up to 3 months post-EDD) and
2. HIE stratum: infants born at ≥ 35 weeks of gestation receiving therapeutic hypothermia for HIE (and up to EDD plus 28 days).

Participating infants will be randomised to receive either the active supplement, or a matched control supplement, plus usual care. Supplementation will commence once infants establish full milk feeds, and continue daily until infants reach 12 months of age post-EDD. Trial data collection will be from trial entry until 24 months post-EDD, including screening, consent, supplementation, and follow-up using case report forms (CRFs). Clinical data is collected from medical records at hospital sites. Participant weight and adherence to daily supplementation during the intervention period will be collected via parent report and/or clinical teams. Additional parental questionnaires will be completed at randomisation and then sent to the parent/carer after discharge and when the child is 3, 6, 12, 18, and 24 months post-EDD. Consent will be obtained to invite parents to participate in future pre-school and school age follow-up studies and to obtain routinely collected education and health data (see Fig. 1 for the trial procedure flow chart).

## Methods: participants, interventions and outcomes

### Study setting {9}

Infants will be recruited from approximately 40 neonatal units (NNU) across the United Kingdom (UK). Participating NNUs will be from the publicly funded UK National Health Service (NHS) and be either of the following:

1. A recruiting NNU where parent consent is obtained in-person or remotely and infants are recruited, randomised, and commence participation in the trial.
2. A continuing care site (CCS), where supplement will be administered by either hospital staff or parents, and trial data collected if a participating infant is transferred in from a recruiting site, or is remotely consented by the recruiting site following transfer.

Following discharge from hospital, supplementation will continue in participants’ homes.

### Eligibility criteria {10}

#### Inclusion criteria

Infants will be eligible for inclusion in the trial if they meet the following criteria:

- ◦ Preterm stratum: Infants born < 28 weeks of gestation, up to discharge to home from hospital (and up to 3 months post-EDD);
- ◦ HIE stratum: Infants born at ≥ 35 weeks of gestation or more, who have received therapeutic hypothermia for HIE (and up to EDD plus 28 days);
- ◦ Individual with parental responsibility able to give consent. In the event that the mother is unable to give consent, or does not have parental responsibility, consent can be given by another person who has parental responsibility. Maternal consent for the purposes of maternal data collection will be sought as soon as practical;
- ◦ Parents able to comply with the protocol;
- ◦ Infants likely to tolerate full enteral feeds;
- ◦ Infant has realistic prospect of survival beyond discharge.

#### Exclusion criteria

The following infants will not be eligible for inclusion in the trial:

- ◦ Infants with middle cerebral artery infarcts;
- ◦ Infants with major congenital brain malformation, or genetic condition with abnormal brain development;
- ◦ Infants with galactosaemia;
- ◦ Infants receiving all feeds via jejunal tube, who do not receive any gastric or oral feeds.

### Who will take informed consent? {26a}

Infants admitted to the NNU will be screened for eligibility by the clinical care team. A trained and delegated staff member in the NNU will approach parents with legal parental responsibility for eligible infants to discuss the trial, request their consent, and complete the consent process as appropriate.

### Additional consent provisions for collection and use of participant data and biological specimens {26b}

Informed consent will be obtained from at least one parent with legal parental responsibility before any trial-related procedures begin. Virtual or remote consent (via telephone or video call) will also be acceptable.

EP infants will be consented either in-person at the recruiting site or remotely after transfer to another hospital. Parental consent for EP infants cannot be obtained after discharge home. Infants with HIE will be consented, either in-person at the recruiting site, remotely after transfer, or after discharge home.

## Interventions

### Explanation for the choice of comparators {6b}

A matched control supplement will be an identically packaged and delivered powder supplement indistinguishable from the active supplement. The control supplement contains smaller amounts of the active components in the investigational product to ensure control supplementation contains nutrients in levels typically obtained through breast milk or infant formula feeding, but no UMP or CMP.

The matched control supplement ensures that any observed effects on cognitive development can be attributed to the active supplement rather than other factors.

### Intervention description {11a}

The intervention is a nutrient supplement powder designed for use with breast milk, infant formula, or food. The active supplement contains a specific blend of nutrients, including LCPUFAs, choline, UMP, CMP, zinc, iodine, and vitamin B12. The active and control supplements are isocaloric, with similar fat content and comparable energy values. The control supplement contains higher levels of lactose. Table 1 shows the amounts of active ingredients in the active and control supplement.

**Table 1:** Amounts of the active ingredients in the active and control supplement (in 1 g powder). *DHA* docosahexaenoic acid, *EPA* eicosapentaenoic acid, *ARA* arachidonic acid, *UMP* uridine monophosphate, *CMP* cytidine monophosphate

	**Active supplement**	**Control supplement**
**Macronutrient composition**		
Energy value (kcal)	5.3	5.1
Carbohydrates (mg)	572.4	607.0
Protein (mg)	89.2	88.0
Fat (mg)	289.7	256.0
Saturates (mg)	104.1	102.0
Monounsaturates (mg)	98.0	113.0
Polyunsaturates (mg)	87.6	41.0
**Active components**		
DHA (mg)	45.1	1.2
EPA (mg)	9.4	0
ARA (mg)	4.8	1.2
UMP (mg)	1.77	0
CMP (mg)	1.77	0
Choline (mg)	10.3	0.3
Zinc (µg)	502.3	11.5
Iodine (µg)	14.8	0.40
Vitamin B12 (ng)	118.2	5.4

Danone Research & Innovation will provide the active and control supplements. Supplementation will start in the neonatal unit once infants reach full milk feeds (120–150 ml/kg/day), or at home for some HIE infants. Supplementation will continue until the infant reaches 12 months post-EDD.

The powdered supplement will be added daily to the infant’s regular milk feed (breast milk, infant formula, or sterile water). It can be administered whilst breastfeeding, bottle feeding, or via a nasogastric/gastrostomy tube. The supplement can also be mixed with weaning foods as the infant transitions to solids. Infants will receive a daily dosage of 1 g of supplement per kg of body weight, up to a maximum daily dose of 12 g. Each 1 g of supplement must be given with a total milk volume of at least 15 ml of milk to ensure appropriate osmolality.

For breastfed babies, 1 g supplement can be mixed with a minimum volume of 3 ml of milk and given before a breastfeed. Alternatively, if a breastfeeding mother is unable to, or prefers not to express milk and does not wish to use infant formula, the supplement can be mixed with a minimum of 3 ml of sterile water per 1 g and administered prior to feeding, as clinically appropriate.

Infants who weigh less than 1 kg will require a daily dose of 0.5 g or 0.75 g of supplement according to defined weight bands and given with a proportionate total feed volume.

Parents will receive training from hospital staff on how to mix and administer the supplement, either in person or remotely, prior to discharge. Upon discharge, families will receive a parent pack containing an initial supply of the supplement, support materials, online video access, dosing instructions, a trial timeline, and contact details for their local NHS clinical team and for the research team.

For infants recruited after discharge, the recruiting site will send the discharge pack. Generic materials, including videos giving step-by-step instructions on supplement preparation for breastfed infants, and for infants receiving the supplement by bottle, nasogastric, or gastrostomy tube are available on the trial website. Specific resources to help promote and protect breastfeeding within the trial are also available on the trial website. These include information sheets on breastfeeding help and support, and supplement use for breastfeeding infants. Table 2 lists trial activities and resources to support participating families.

**Table 2:** Trial activities and resources to support participating families

Trial phase	Activity or resource
**Pre-application patient and public involvement (PPI) and engagement**	Engagement with parent participants from the Dolphin Neonatal and Infant Trials (24–26) to explore experiences of giving the nutritional supplement in breast milk, infant formula milk, and solid foods
**Trial design including focus on the protection and promotion of breast feeding**	Engagement with national and international breastfeeding support groups and initiatives to ensure that trial design protects and promotes breastfeeding within the trial Consultation with infant feeding specialists and the DOLFIN parent advisory group (PAG) about safe and effective supplemental feeding system use for breastfeeding infants and sites provided with funding for these Provision of breast milk pumps for parents at sites Bench testing of nutritional supplement in breast milk to determine supplement solubility in a small volume breast milk shot Creation of videos demonstrating how to prepare and administer the supplement to breastfed, infant formula-fed and tube-fed infants Sign-posting to breastfeeding support organisations on trial website Collection of data on breastfeeding rates and duration amongst parent participants via parent questionnaires
	Parent co-applicants who have been involved in the application for funds, trial design and delivery PAG input to parent-facing trial materials including Parent Information Sheets and Parent Information for Supplement Use documents Bliss (national neonatal charity) co-applicants who have been involved in the application for funds, trial design and delivery
	Creation of ‘Easy Read’, short information sheet versions of key trial documents to maximise recruitment of people with additional needs and/or learning disability Development of a parent trial app to support collection of adherence and safety information
**Recruitment**	Consultation with Southeast Asian community groups to explore barriers to recruitment specific to this community Engagement and partnership with Peeps HIE charity with signposting to parents for support Materials for parents on NNUs—flyers with short information and QR codes signposting to materials
**Retention**	Creation of parent newsletters showing evidence about recruitment and giving key trial messages Delivery of parent webinars focusing on key trial messages and how parents can support their child’s early development Creation of parent participant videos to help parents understand what taking part in the trial involves Sharing of neurodevelopmental outcome measures with local NHS clinical team to support clinical care and parent knowledge Creation of a parent video explaining the importance of 24-month questionnaire completion. Link to video embedded in questionnaire completion request Introduction of telephone appointment booking system giving parents option to complete the 24-month questionnaire via telephone Option of 24-month questionnaire completion during a home visit for parents who require additional support
**Dissemination**	PAG involvement in planning of trial results dissemination activities Written research finding summary for parents Parent webinar summarising trial findings Summaries for charity websites

During the supplementation period, parents will be asked to submit their infant’s weight monthly through the trial app or via a link to a CRF sent by text or email, or on a paper version if preferred. To support parents increasing the supplement dose according to infant weight, the app and form will reference the dosing schedule for each weight band that is provided as a paper document at discharge, and online via the trial website.

The research team includes infant feeding specialists, a speech and language therapist, a dietitian, paediatricians, and neonatologists. These professionals will provide feeding and supplementation advice to local NHS clinical teams. The infant feeding specialists provide support and advice to local NHS clinical teams in relation to breastfeeding within the trial.

### Criteria for discontinuing or modifying allocated interventions {11b}

Parents will have the right to change their consent for their infant’s participation in the trial at any time (withdrawal). Change of consent will not affect the infant’s ongoing clinical care. They may change consent for any aspect of the trial and/or data collection. Data collected up to the point of change of consent will be used in the trial.

Where parents choose to permanently discontinue the trial supplement, they will be asked if they continue to agree to complete data collection and to give permission for the trial team to complete data collection using their medical records. The treating clinician may discontinue the trial supplement if deemed necessary for the infant’s health and well-being.

### Strategies to improve adherence to interventions {11c}

To provide information about adherence to interventions, sites will record data on dosing during the infant’s hospital stay using a CRF. Post-discharge, parents administering the supplement will enter data about whether or not the supplement has been given, using either the bespoke trial app, an OpenClinica CRF, or a paper diary, according to preference. Further details about the DOLFIN app can be found in Data collection and management (Sect. 18a). For the first month post-discharge, parents will receive daily reminders via the trial app, email, or text asking them to confirm each day that they have given the supplement, with the option to adjust reminders if preferred. After the first month, weekly prompts will be sent to report the proportion of supplement given. Parents can opt to continue daily reminders if they prefer. In instances of non-adherence, the research team will work with the local NHS clinical team to understand any barriers to supplementation and provide appropriate support to enhance adherence.

Until 6 months post-EDD, a research nurse from the recruiting site will make monthly calls to address any parental queries regarding supplementation and to confirm the correct dose is being administered. Calls will cease once parents are confident in the dosing process.

### Relevant concomitant care permitted or prohibited during the trial {11d}

There are no contraindicated medications or dietary supplements, and infants will be able to have all medicines and supplements normally prescribed for this population during the trial. If parents choose to give additional dietary LCPUFA to their infant, this is not a concern, as commercially available LCPUFA supplements are low dose and will not significantly alter the overall LCPUFA intake of participating infants.

### Provisions for post-trial care {30}

Not applicable. Given that the intervention poses minimal risk to participants, this trial protocol does not include provisions for ancillary or post-trial care, nor for compensation in the event of harm resulting from participation.

### Outcomes {12}

#### Primary outcome>

The primary outcome measure is the non-verbal cognitive scale standardised score of the PARCA-R questionnaire [30] at 24 months post-EDD. The PARCA-R is a reliable and validated parent completed norm-referenced assessment from which standard scores are derived for cognitive and language development [30]. The PARCA-R non-verbal cognitive subscale comprises 34 items for which the parent is asked to respond ‘yes’, ‘no’, or ‘don’t know’ to whether their child has exhibited a specific ability. The number of ‘yes’ responses are summed to produce a non-verbal cognitive subscale raw score (range 0 to 34). The raw score is converted to an age-standardised score with a normative mean of 100 [SD 15], with higher PARCA-R scores indicating lower degrees of impairment [30]. PARCA-R was used as an outcome measure in recent landmark perinatal trials (Speed of Increasing Milk Feeds Trial (SIFT), International Neonatal Immunotherapy Study (INIS), Computerised interpretation of fetal heart rate during labour (INFANT)), and is recommended for developmental surveillance of children born EP at 24 months of age corrected for prematurity [31, 32].

#### Secondary outcomes

The following outcomes will be assessed at 24 months post-EDD unless otherwise stated:

1. Tested (i.e. statistical inference made):
  ◦ Language scale standardised score of the PARCA-R questionnaire. The PARCA-R language subscale comprises a 100-word vocabulary checklist, from which the number of words the child can say is summed to produce a score (range 0 to 100), along with 18 forced-choice items about the child’s use of sentences (score range of 0 to 24). These are summed to produce the language subscale raw score (range 0 to 124). The raw score is converted to an age-standardised score, as per the non-verbal cognitive subscale;
  ◦ Parent reported emotional, conduct, peer problems, hyperactivity, prosocial, and total score using the Strengths and Difficulties Questionnaire (SDQ) [33]. Each SDQ component contains 5 statements to which the parent responds as ‘not true’, ‘somewhat true’, or ‘certainly true’. Each response has a maximum possible score of 2; each component has a maximum score of 10. A lower score indicates a lower level of difficulties. The overall SDQ score is calculated by summing each of the components;
  ◦ Parent reported motor skills using the fine and gross motor scales score of the Ages and Stages Questionnaire 3rd Edition (ASQ-3) [34]. Each scale consists of questions which the parent reports as ‘yes’ (10 points), ‘sometimes’ (5 points), or ‘not yet’ (0 points). Responses are summed to give the scale score. Each scale has a maximum possible score of 60, with a higher score indicating less concern about developmental status.
  ◦ Weight standard deviation (SD) score, (z-score), is calculated using the World Health Organization (WHO) child growth standards charts [35] which standardises growth by age, sex, and country;
  ◦ Head circumference SD score, (z-score), is calculated using the same process as the weight SD score [36];
  ◦ Overweight or obese (defined as BMI ≥ 85th percentile) according to UK90 growth reference data [37];
  ◦ Microbiologically-confirmed late-onset invasive infection up to discharge home from the neonatal unit;
  ◦ Necrotising enterocolitis requiring surgery up to discharge home from the neonatal unit;
  ◦ Retinopathy of prematurity treated medically/surgically in the preterm stratum only, up to discharge home from the neonatal unit;
  ◦ Chronic lung disease (oxygen requirement at 36 weeks corrected age) in the preterm stratum only, up to discharge home from the neonatal unit;
  ◦ Maternal health-related quality of life, and associated utility, as measured using the EuroQoL EQ-5D-5L questionnaire [38] at 6, 12, 18, and 24 months post-EDD. The EQ-5D-5L is designed for self-completion by respondents [39]. Responses will be converted into utilities using tariffs estimated from a representative UK population sample [40]. Infant secondary care resource use and costs up to 24 months, which will be extracted from site hospital records.
  ◦ Infant health care data (to capture health care utilisation data not available from hospital records), social care resource use and costs associated with any major specialist items purchased by families, or home adaptations for infant care as measured by the Bespoke Health Economic questionnaire will be completed at 6, 12, 18, and 24 months post EDD; Productivity costs and informal care up to 24 months post-EDD. Changes in parents’ work patterns or time away from work, and any additional informal care/support required;
  ◦ Incremental cost per life year gained without moderate/severe neurodevelopmental impairment;
  ◦ Incremental cost per quality-adjusted life year (QALY) gained modelled to 18 years post-EDD.
2. Untested
  ◦ Safety and adverse events up to 12 months plus 2 weeks after the end of the intervention period, including all serious adverse events (SAEs)
  ◦ Parent reported infant tolerability of supplement (IGSQ-13) [41] measured at discharge home from the neonatal unit, and at 3, 6, and 12 months post-EDD. The IGSQ Index is a 13-item questionnaire where parents report infant’s recent gastrointestinal symptoms across 5 domains (stooling, spitting up/vomiting, crying, fussiness, and flatulence). Each item is given a score between 1 and 5, with a score of 1 indicating no gastrointestinal symptoms. Overall IGSQ score is calculated by summing the item scores, (range 13 to 65). A higher score indicates greater symptom-related distress.
  ◦ Parent reported adherence measured up to 12 months post-EDD
  ◦ Parent reported acceptability of supplement at 6 and 12 months post-EDD
  ◦ Fit or seizure in the past 12 months, measured up to 24 months post-EDD
  ◦ Presence of feeding tube of any type, up to 24 months post-EDD
  ◦ Receiving supplemental oxygen or any respiratory support, up to 24 months post-EDD 
  ◦ Confirmed or suspected diagnosis of cerebral palsy, up to 24 months post-EDD
  ◦ Hydrocephalus treated with a third ventriculostomy or ventriculo-peritoneal shunt, up to 24 months post-EDD

### Participant timeline {13}

**Fig. 2:**
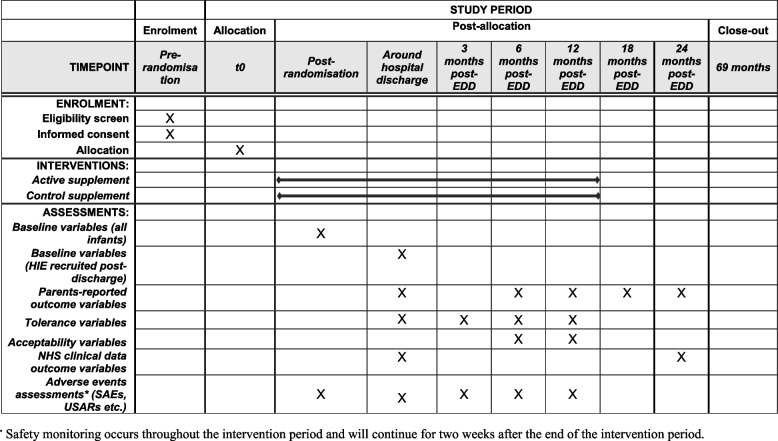
SPIRIT figure displaying the schedule of enrolment, interventions, and assessments

SPIRIT figure displaying the schedule of enrolment, interventions, and assessments (see Fig. 2).

### Sample size {14}

To detect a 6-point difference between the two trial arms on the PARCA-R non-verbal cognitive scale standard score (for both strata), with 90% power and a two-sided 5% significance level, assuming a population mean score of 88 and standard deviation of 19, 212 infants per arm are required for each stratum (424 in the preterm stratum and 424 in the HIE stratum). The estimated mean and standard deviation are from infants born less than 28 weeks of gestation in the Perinatal and Neonatal Data Analysis (PANDA) study (used as validation sample for PARCA-R standardisation) [42]. We assume similarly for infants of ≥ 35 weeks of gestation with HIE. An inflation factor of 14% applied to the preterm stratum allows for clustering due to infants from multiple births being randomised to the same allocation, giving a total of 484 infants (assuming prevalence of multiples of 30% and intra-cluster correlation coefficient of 0.77, data from SIFT trial) [43]. Prevalence of multiple births in the HIE stratum is expected to be negligible. Allowing for 10% loss to follow-up at 2 years of age gives an overall sample size of 538 (269 per arm) in the preterm stratum and 472 (236 per arm) in the HIE stratum, giving an overall total sample size of 1010 infants. Infants who have died, or for whom a change of consent has been received where this results in discontinuation of supplement and/or little to no data collection, within 28 days of commencing the supplement, will be replaced through additional trial recruitment.

Each stratum of the trial was powered to detect a clinically relevant 6-point difference in the PARCA-R cognitive scale score. In previous studies of EP infants a 6-point increase in cognitive test scores (Bayley Scales of Infant Development II Mental Development Index) at 2.5 years of age (corrected for prematurity) was associated with a 3.5-point (95% CI, 2.3 to 4.7) increase in IQ test scores at 19 years of age (Wechsler Abbreviated Scale of Intelligence, second edition (WASI-II) Full Scale IQ) (*n* = 115), and a 13% reduction in risk of having SEN at 11 years of age (risk ratio (RR) 0.87, 95% CI, 0.84 to 0.90, *n* = 190).^1^ If the DOLFIN trial identified a 6-point difference in cognitive test score between intervention and control group infants, the IQ advantage would be comparable to that conferred by breastfeeding [44], which has driven international practice change to strongly promote breastfeeding.

### Recruitment {15}

Recruiting sites will conduct regular screening for eligible infants by reviewing admission and discharge lists. Screening and eligibility checks will be carried out by trained staff, with final eligibility confirmed by a delegated individual. Parents of potentially eligible infants will be approached either in person or remotely to discuss trial participation, and sites will ensure parents are informed about the recruitment window. If infants are transferred or discharged before a decision is reached, sites will follow up as needed. Infants in the preterm stratum must be recruited prior to discharge from hospital, either at a recruiting site or at a CCS via remote consent. Infants in the HIE stratum can be recruited in hospital, or from home following discharge. Trial materials such as posters, banners, and leaflets will be displayed in neonatal units to raise awareness among staff and families. Co-recruitment to other trials is permitted, except for intervention trials which have a neurodevelopmental primary outcome. Co-recruitment to another trial with a neurodevelopmental primary outcome may be possible following discussion and agreement between trials chief investigators (CI).

## Assignment of interventions: allocation

### Sequence generation {16a}

The randomisation system used to allocate participants in DOLFIN uses a bespoke system developed using PHP programming language and a MySQL database and is managed by the National Perinatal Epidemiology Unit (NPEU) Clinical Trials Unit (CTU) at the University of Oxford, with telephone backup available at all times (365 days per year). Participants are randomly assigned in a 1:1 allocation ratio to either the active supplement or the matched control supplement. Randomisation is stratified according to the two strata: (1) EP infants born < 28 weeks of gestation (and up to 3 months post-EDD); and (2) infants born ≥ 35 weeks of gestation who receive therapeutic hypothermia for HIE (and up to 28 days post-EDD). The randomisation program uses a separate minimisation algorithm within each stratum to ensure balance between the intervention and control arms with respect to recruiting hospital and sex, and in addition for the preterm strata, gestational age at delivery (by week of gestation) and multiple births. Multiple births (e.g. twins or triplets) are assigned to the same intervention arm.

### Concealment mechanism {16b}

A Senior Trials Programmer at the NPEU CTU wrote the web-based randomisation programme and will hold the treatment allocation codes. Pack numbers will be added by the Senior Trials Programmer, who will liaise directly with the packaging and distribution company. The Senior Trials Programmer and Trial Statisticians will monitor implementation of the randomisation procedure throughout the trial. Reports on the randomisation balance across the study strata will be provided to the Data Monitoring Committee (DMC) (see section on Interim Analyses). An integrated web-based pack management system will track supplies of active supplement and control supplement ensuring a balance of stock across sites.

### Implementation {16c}

Staff at recruiting sites will log in to the randomisation system using a unique site login to randomise participants. The system will confirm eligibility, assign a unique DOLFIN trial identification, and link each infant to a treatment pack containing sachets of either the active supplement or control supplement. A backup telephone randomisation service will also be available.

## Assignment of interventions: blinding

### Who will be blinded {17a}

Families and clinical teams caring for the infant, as well as all investigators and CTU staff, will be blinded to the trial allocation. Only the Senior Trials Programmer and Trial Statisticians will have access to this information.

### Procedure for unblinding if needed {17b}

In the event of an emergency, unblinding can be conducted by the clinician at the recruiting site using a single-use access code from a sealed envelope to log in to the randomisation website. The reason for unblinding must be recorded in the randomisation database, and clinicians will be reminded to exercise caution in handling unblinded information. Clinicians must be certain that unblinding is essential for managing the emergency and that knowledge of the treatment allocation is necessary for appropriate care. Clinicians are encouraged to consult with the site Principal Investigator or other delegated clinicians before unblinding, if possible and safe to do so. If the infant has been transferred to another site, the treating healthcare professional should contact the PI or any delegated clinician at the recruiting site to discuss unblinding.

Since long-term neurodevelopmental follow-up is planned, participants will not be unblinded until future follow-up studies are complete. Any unblinding requests prior to this must be made in writing to the NPEU CTU, who will discuss the request with the CI (or their delegate). The unblinding process and criteria will be communicated to participants in the Parent Information Sheet prior to obtaining consent.

## Data collection and management

### Plans for assessment and collection of outcomes {18a}

Data collection for the DOLFIN trial will encompass the completion of electronic CRFs by both site staff and parents throughout the trial period. Site staff will primarily enter data electronically through the randomisation site and OpenClinica, while any manually collected data will be entered into OpenClinica at a later stage. Manually collected data will be securely stored in a locked cabinet and accessible only to authorised personnel prior to being entered into the electronic database. In addition, parents will be invited to download a bespoke trial app, created in partnership with NuTH, and produced by Newcastle University, to facilitate collection of adherence and safety data, as well as communication from the research team. The app is not a medical device and does not calculate supplement dosage for participants.

The key CRFs include consent forms, which must be completed by both parents and site staff, either in person or remotely, prior to the initiation of any data collection. After obtaining consent, randomisation forms will be completed via the DOLFIN randomisation website. Site staff will be responsible for completing the entry form, with additional baseline data, upon enrolment. When the infant begins receiving the supplement, the Supplement Starter Form will be filled out, and a Dosing Log maintained to track daily supplement adherence for doses administered in hospital. Adherence recording will be continued by parents post-discharge using the app.

In instances where an infant is transferred between hospitals, Transfer/Discharge Forms will be required. Upon discharge, whether from the recruiting site or a CCS, site staff will complete a discharge form, with assistance from CCS staff as necessary.

Parents will receive electronic links to complete questionnaires at baseline, upon discharge, and at 3, 6, 12, 18, and 24 months post-EDD. To accommodate parents’ preferences, paper versions will also be available.

### Plans to promote participant retention and complete follow-up {18b}

During the first 6 months of their trial participation, parents will receive monthly phone calls from the local research nurse to confirm the appropriate supplement dose based on the infant’s weight and to provide advice on any parental supplementation queries. These calls will be discontinued once parents are confident in dosing, and confirm they no longer wish to receive a regular phone call.

An electronic communication (e.g. via the trial app, email, WhatsApp, text message) will also be used to prompt parents to complete and return questionnaires at randomisation, around discharge from NNU, and 3, 6, 12, 18, and 24 months post-EDD. A pre-paid envelope will be supplied to families who opt to complete paper copies of the questionnaires. Participants who do not complete the outcome measures are contacted with a further request for completion. Thereafter, failure to complete the outcome questionnaires will trigger contact from a member of the local NHS clinical team or from the research team to check if there are any barriers to completion and offer support in completing the questionnaires. Support may include, for example, an offer to complete the questionnaire over the phone at a time convenient to the family. A home visit from the local research nurse may be offered if this is necessary to ensure the completion of the primary outcome measure. For parents who remain uncontactable, a letter will be sent including a paper copy of the last outstanding questionnaire. Following completion of the 24 month questionnaire, primary outcome (PARCA-R) and secondary outcome neurodevelopmental data will be shared with the local neonatologist or paediatrician, or general practitioner for participants no longer under hospital follow-up; information about norms will be provided to aid local clinician interpretation. Participating families will be sent a £25 gift voucher as a thank you for their participation in the trial at the time that 24 months parent-completed outcome measures are requested. Where a response to the 24 months follow-up questionnaire has not been received or the questionnaire is incomplete after the above data collection steps have been followed, the PARCA-R may be obtained from clinical records where this has been recorded during routine medical visits.

To support ongoing engagement, parents will also be invited to parent webinars and receive newsletters with updates on trial progress.

### Data management {19}

The data management aspects of the trial are fully described in the Data Management Plan to ensure that high quality data are produced for statistical analysis.

Source documents are where data are first recorded, and from which infants’ CRF data will be obtained. CRF entries are considered source data if the CRF is the site of the original recording. Parent-reported data (for example, adherence data collected via the app, Quality of Life data and 24-month questionnaires) are considered source data.

Authorised representatives from the sponsor, funder, research team, NHS trust, and regulators have direct access to data for monitoring and auditing. Site staff have restricted, password-protected access to the clinical database. There will be no direct sharing of patient or professional details with Danone Research & Innovation. Data shared with Danone Research & Innovation will be anonymised or pseudo-anonymised, in line with the Product Supply Agreement (see Interim Analyses Sect. 21b).

Clinical data will be entered into a validated database, with validation checks for accuracy. Participant names and identifiable information will be stored in a separate administrative database. Consent forms will be sent securely, and personal data protected according to Standard Operating Procedures (SOP). The app developed during the trial will store minimal personal details for app usage and will not share them externally.

Electronic and paper records are stored securely, with restricted access to authorised personnel. Electronic files are stored on a secure server, with regular backups and restricted entry. Archiving of the Trial Master File will follow the requirements of NPEU CTU SOPs and applicable NHS guidelines; data will be archived for at least 25 years, with a review of further archiving needs based on data protection laws.

At the end of the trial, all participant data will be transferred to Newcastle University and Newcastle upon Tyne Hospitals NHS Foundation Trust for long-term follow-up, in compliance with data sharing agreements.

### Confidentiality {27}

The trial will comply with the General Data Protection Regulation and the Data Protection Act 2018. All documents will be stored securely at the NPEU CTU and will only be accessible by trial staff and authorised personnel. The trial staff will safeguard the privacy of participants’ personal data.

All personal identifier details and trial data will be stored in separate databases held at the NPEU CTU, linked by the infant’s trial number. After trial completion, a separate database of personal identifier details and trial data will also be held at Newcastle University and Newcastle upon Tyne Hospitals NHS Foundation Trust.

After the trial has been completed and the reports published, the data will be archived in secure physical or electronic locations with controlled access.

### Plans for collection, laboratory evaluation and storage of biological specimens for genetic or molecular analysis in this trial/future use {33}

Not applicable. This trial protocol does not include the collection, laboratory evaluation, or storage of biological specimens for genetic or molecular analysis, either for the current trial or for future ancillary studies.

## Statistical methods

### Statistical methods for primary and secondary outcomes {20a}

#### Baseline characteristics

Socio-demographic characteristics of the mothers, and clinical characteristics of the infants will be reported by trial allocation. For binary and categorical variables, the number and percentage in each category will be presented. For continuous variables, the mean and standard deviation or the median and interquartile range will be presented. There will be no tests of statistical significance for differences between randomised groups on any baseline variable.

#### Primary outcome

Statistical analyses will be conducted according to a pre-specified statistical analysis plan (SAP), which will be available in a separate document. The SAP will provide detailed information on the methods used to analyse the primary and secondary outcomes, including any adjustments for covariates and multiple comparisons.

Data will be analysed separately for the two patient populations/strata. The primary analysis will use a modified intention-to-treat (ITT) approach, meaning infants with outcome data available will be analysed based on their random allocation, regardless of the treatment received.

For the primary outcome of PARCA-R non-verbal cognitive scale standard score at 24 months post-EDD, the mean and standard deviation will be presented by randomised group. For infants whose PARCA-R questionnaires are completed outside 23.5 to 27.5 months post-EDD, standard scores cannot be calculated, but raw scores may be available. A multiple imputation analysis will estimate standard scores for these infants, with these estimates presented as the primary inference. For comparative analysis, a mixed-effects linear regression model will be fitted on the multiply imputed datasets. The models will be adjusted for minimisation factors, which include recruiting hospital and sex in the HIE stratum, and recruiting hospital, sex, gestational age at delivery (by week), and multiple births in the preterm stratum. The recruiting hospital will be treated as a random effect, while all other minimisation factors will be treated as fixed effects. The correlation between infants from multiple births will be accounted for by nesting multiple births as a random effect within the recruiting centre. If this approach is not technically possible, clustering for multiple births will be included in the model with recruiting centre treated as a fixed effect. Adjusted mean standardised scores, adjusted mean differences, and 95% CI will be presented.

#### Secondary outcomes

Secondary outcomes will also be analysed and presented separately for each stratum and will use the modified ITT approach.

For dichotomous outcomes, risk ratios and associated 95% CIs will be estimated using a log-binomial regression model, or alternatively a Poisson regression model with a robust variance estimator (if the binomial model fails to converge). For continuous outcomes, mixed-effects linear regression models will be applied, with mean differences and associated 95% CIs. If the data show significant skewness, quantile regression will be used, and median differences along with associated 95% CIs will be presented. Growth measurements, such as weight and head circumference, will be converted to standard deviation scores, with analyses adjusted for baseline growth score.

Models used for the analysis of secondary outcomes will follow the same approach as for the primary outcome. The correlation between infants from multiple births will be accounted for by nesting multiple births as a random effect within the recruiting centre. If this approach is not technically possible, clustering for multiple births will be included in the model with recruiting centre treated as a fixed effect.

Secondary outcomes that are not tested will be analysed descriptively, as per the descriptive analysis of baseline characteristics. No tests of statistical significance will be performed, and no confidence intervals will be calculated between the randomised groups for the untested secondary outcomes.

#### Health economics analysis plan (HEAP)

A cost-effectiveness analysis to determine whether the potential benefits of the nutritional supplement represent value for money will be conducted separately for the EP and HIE strata. A HEAP with extended details of the costs-effectiveness methods presented in the protocol will be prepared in a separate document. A two-stage economic evaluation will be conducted to assess: (1) whether the resources needed to deliver the nutritional supplement in practice are justified by the benefits achieved at 24 months; and (2) to estimate the cost-effectiveness of the nutritional supplement up to 18 years of age.

In the first stage, a within-trial cost-effectiveness analysis to assess the incremental cost per life year gained without moderate/severe neurodevelopmental impairment at 24 months will be undertaken. All analyses will be conducted from both an NHS health, social care, and societal perspective. To minimise burden to families, secondary care data will be extracted from hospital records at each site. Parents will receive electronic links to complete questionnaires when infants are 6, 12, 18, and 24 months post EDD to capture health care utilisation data not available from hospital records (i.e. primary and community health and social care usage), any major specialist items purchased by families, or home adaptations for infant care; changes in parents’ work patterns or time away from work, and any additional informal care/support required. Costs of the intervention will also include those associated with delivery of the intervention to both the NHS and families, with a micro-costing trial used to determine these costs. All resource use will be assessed using average unit costs from established national sources such as NHS Reference Costs and the Personal and Social Services Research Unit cost compendium [45]. Mean incremental analysis of costs and life-years without moderate/severe impairment in the active compared to the control supplement groups will be synthesised using the incremental cost-effectiveness ratio (ICER), which will be expressed as cost per life years without moderate/severe neurodevelopmental impairment gained. Uncertainty around that estimate will be presented using parametric and non-parametric CI for the ICER (if appropriate) and cost-effectiveness acceptability curves (CEAC).

For the within-trial economic evaluation, a secondary analysis will be reported whereby results will be presented as a cost-consequence analysis, i.e. costs and outcomes will be presented in a disaggregated manner. As part of this analysis, maternal EQ-5D-5L quality of life data will be presented as maternal QALYs gained over the 24-month trial period.

In the second stage, if the nutritional supplement is shown to be effective for either strata, a decision analytical model to estimate the cost-effectiveness of the intervention up to 18 years of age will be developed. This analysis will be conducted from an NHS and societal perspective. The main outcome measure in the economic evaluation will be child QALYs. A Markov model will be constructed representing the natural history of infants born < 28 weeks of gestation and term infants with HIE who receive therapeutic hypothermia to extrapolate the within-trial cost-effectiveness results using annual cycles. The structure of the model will be established and agreed within the research team. Observed outcomes and health care resource utilisation for randomised infants will be used to inform the characteristics of a hypothetical cohort entering the model. Transition probabilities indicating movement across health states during the first 2 years will be obtained from the trial, whereas transition probabilities after the second year will be informed through literature searches. Health care costs and health-related quality of life estimates incurred annually in each health state after the second year will be obtained from the literature. Data on informal care and impact on productivity of parents to inform cost parameters in the model will also be obtained from the literature if available.

Costs and QALYs will be combined and synthesised using the ICER and the net-benefit statistic. Uncertainty will be assessed using probabilistic sensitivity analysis and CEACs. One-way sensitivity analysis will be carried out to explore the impact of parameters not subject to probabilistic uncertainty (e.g. cost of active supplement to the NHS) on cost-effectiveness results.

#### Interim analyses {21b}

The DMC, independent of the applicants and Trial Steering Committee (TSC), will review the progress and accumulating data from the trial at least annually and provide advice on the conduct of the trial to the TSC. DMC meetings will include open and closed sessions. The open session will cover recruitment, data quality and pooled safety data. The closed session will focus on accumulating efficacy and safety data by trial arm, reported using summary statistics. There are no planned comparative interim analyses; *p*-values and confidence intervals will only be calculated and presented if requested by the DMC in response to safety or efficacy concerns.

### Methods for additional analyses (e.g. subgroup analyses) {20b}

Pre-specified subgroup analyses will assess the consistency of the supplement’s effect on the primary outcome across specific infant subgroups, using statistical tests of interaction. These subgroups include gestational age by week for EP infants, and the severity of HIE (normal/mild, moderate, severe) according to neurological examination on days 1, 2, and 3 of therapeutic hypothermia for the HIE patient population. Effect estimates and 95% CI will be presented for each subgroup, plus the interaction *p*-value.

Sensitivity analysis will be carried out excluding infants with imputed PARCA-R scores in the primary analysis. Additional sensitivity analyses will be conducted excluding (a) infants who started supplementation more than 28 days post-randomisation, (b) those who met exclusion criteria post-randomisation (e.g. with cerebral infarcts, congenital brain malformations or a genetic condition with abnormal brain development, or galactosaemia), and (c) infants receiving exclusive jejunal feeds.

### Methods in analysis to handle protocol non-adherence and any statistical methods to handle missing data {20c}

Adherence to the trial intervention will be reported by trial arm for each of the patient populations. Missing data will be reported by showing the number of individuals with missing information for all outcomes. For the primary outcome, as outlined earlier, infants with questionnaires completed outside the 23.5- to 27.5-month age range will have their raw scores imputed using multiple imputation. In addition, for infants where four or fewer questions for the non-verbal cognitive scale are missing, the mean score of their completed questions will be imputed for these missing questions. Where more than four questions are missing, a non-verbal cognitive score cannot be derived and these infants will be treated as missing. The dataset which includes the substituted scores where four or fewer questions are missing and the multiple imputation estimates will be used for the primary analysis. No further imputation of missing data will be carried out.

### Plans to give access to the full protocol, participant level-data and statistical code {31c}

Prior to the recruitment of the first participant, the trial will have been registered on a publicly accessible database. The trial information will be kept up to date during the trial, and the CI or their delegate will upload results to all those public registries within 12 months of the end of the trial declaration. All data requests should be submitted to the corresponding author of the main results paper for consideration. Access to de-identified patient data may be granted for secondary analysis following review. If approved, a data sharing agreement will be put in place. Data availability will begin with publication of the final results.

## Oversight and monitoring

### Composition of the coordinating centre and trial steering committee {5d}

The trial will be run on a day-to-day basis by the Project Management Group (PMG), which reports to the Trial Steering Committee (TSC), which in turn is responsible to the National Institute for Health and Care Research Health Technology Assessment (NIHR HTA) programme (as per the NIHR HTA contract). The PMG will consist of the CI, CTU Director, Clinical CTU Director, Senior Trials Manager, the Trial Statistician, Sponsor, and other project staff. The PMG will meet every month. The Co-Investigator Group, will comprise all members of the co-applicant group and the members of the PMG to review progress, troubleshoot and plan strategically. The trial will be overseen by a TSC consisting of an independent chair and other members to include clinicians, statisticians, and PPI representatives. Committee members will be deemed independent if they are not involved in trial recruitment. The TSC will aim to meet in person or virtually at least annually. The TSC will monitor the progress of the trial and its conduct and advice on its scientific credibility. The TSC will consider and act, as appropriate, upon the recommendations of the DMC and ultimately carry the responsibility for deciding whether the trial needs to be stopped on grounds of safety or efficacy.

### Composition of the data monitoring committee, its role and reporting structure {21a}

The DMC members are independent of the trial team and the TSC, and include a chair, clinician, and statistician. During the recruitment phase, the committee will meet annually or more often as appropriate, to review trial conduct, progress and accumulating data, and make recommendations to the TSC. Details about the roles, responsibilities, and conduct of the committee will be set out in a DMC Charter, which will be agreed at the first meeting.

### Adverse event reporting and harms {22}

Safety monitoring for each participant will begin with the administration of the supplement and continue for 2 weeks after the trial supplement period, which lasts until 12 months post-EDD. In this population, we anticipate day-to-day fluctuations of pre-existing conditions, new conditions, and a small number of deaths. As a result, many adverse events are foreseeable due to the nature of the participant population and their routine care/ treatment. Consequently, only those adverse events identified as serious will be reported for the trial. The protocol provides detailed information on all foreseeable SAEs within the trial population that do not require reporting, ensuring that this information is accessible to all participating sites. Foreseeable SAEs do not need to be reported unless there is concern of a causal link to the trial supplement.

SAEs not listed as foreseeable and not deemed causally related will be reported. Specific events requiring reporting include the following:

- ◦ Serious, prolonged gastrointestinal disturbances (excluding necrotising enterocolitis)
- ◦ Such disturbances associated with culture/growth of unusual organisms
- ◦ Sepsis with culture/growth of unusual organisms

Any unforeseeable SAEs or those deemed to be causally related to the trial supplement must be reported to NPEU CTU as soon as possible after it is defined as serious. A medically qualified site staff member will assess the relationship of each serious adverse event to the trial supplement.

Unexpected Serious Adverse Reactions (USARs) will be reported to the REC that gave favourable opinion, within 15 working days. Additionally, a copy of the SAE form related to the event will be forwarded to the Chair of the DMC for their review.

The DMC oversees the review of trial data and safety reports pertaining to SAEs. The DMC is charged with ensuring the safety and well-being of trial participants and will make recommendations to the TSC regarding the continuation of the trial or necessary modifications to the protocol. The TSC holds the final authority to determine whether the trial should be terminated for safety reasons.

### Frequency and plans for auditing trial conduct {23}

The PI will oversee the trial at their site, which includes ensuring effective recruitment, providing staff education and training, and maintaining the completeness and quality of trial data.

The NPEU CTU will establish a comprehensive central monitoring plan for the trial based on the risk assessment. Recruitment patterns at the sites, as well as within the data, will be closely monitored. Any unexpected patterns, issues, or outlier data will be thoroughly investigated and may prompt targeted site monitoring. Routine monitoring or auditing is not conducted unless the central monitoring identifies a specific need for it.

### Plans for communicating important protocol amendments to relevant parties (e.g. trial participants, ethical committees) {25}

The NPEU CTU will submit and, where necessary, obtain approval from the Sponsor, REC, HRA, and host institution for written approval for all substantial amendments to the original approved documents.

## Dissemination plans {31a}

In addition to Protocol publication, articles reporting key trial outcomes will be submitted to open access peer reviewed journals for publication. Trial findings will also be presented at national and international conferences. The DOLFIN investigators will disseminate the trial results and coordinate press releases, trial website promotion, and social and other media interest. Parents will be emailed a copy of the trial results.

## Discussion

This is the first randomised controlled trial to assess whether combined nutritional supplementation with LCPUFAs, choline, UMP, CMP, zinc, iodine, and vitamin B12 plus usual care from birth to 12 months post-EDD improves neurodevelopmental outcomes at 24 months post-EDD, compared to infants receiving a matched control supplement plus usual care, in infants born EP, or born at ≥ 35 weeks of gestation and receiving therapeutic hypothermia for HIE. A similar trial in the Netherlands is currently assessing the effect of the same nutritional supplement on brain white matter development in EP-born infants using magnetic resonance imaging techniques, and includes a range of secondary neurodevelopmental outcomes [46].

Mechanisms of brain injury vary between EP and HIE infants. In infants born EP, brain injury is characterised by white matter injury, whereas in term HIE, injury is predominantly to short cortico-cortical connections and to areas of high metabolic activity, with subsequent secondary and tertiary injury mediated via inflammatory processes. These differences in mechanisms of brain injury are addressed by undertaking a trial stratifying EP and HIE infants, each powered to detect a 6-point difference in the nonverbal cognitive scale score of the PARCA-R at 24 months post-EDD (primary outcome measure), and each stratum analysed separately.

If the DOLFIN trial shows improved cognitive outcome following nutritional supplementation of EP infants and HIE infants who received therapeutic hypothermia, this intervention could improve cognitive outcome for millions of infants worldwide, and inform international nutritional practice and usual clinical care for vulnerable infants. Trial findings will also contribute valuable insights into the importance of nutritional optimisation for brain development following perinatal brain insult.

## Trial status

Protocol version V6.0, dated 10/10/2024. At the time of submission of the original manuscript, participant recruitment and enrolment for the DOLFIN trial was ongoing. Recruitment commenced on 1st October 2022, and ended on 15th April 2025. Data collection is ongoing and will complete in winter 2026.

## Data Availability

Data requests should be directed to the corresponding author for review and consideration. Please be aware that exclusive access to the data will be maintained until after publication of the main trial findings. After this, access to anonymised data may be granted following a detailed review process.

## Abbreviations

ARA: Arachidonic acid
ASQ-3: Ages and Stages Questionnaire 3rd Edition
BMI: Body mass index
BPD: Bronchopulmonary dysplasia
CCS: Continuing care sites
CEAC: Cost-Effectiveness Acceptability Curves
CI: Confidence Interval
CI: Chief investigator
CMP: Cytidine-5′-monophosphate
CRF: Case report form
CTU: Clinical Trial Unit
DHA: Docosahexaenoic acid
DMC: Data Monitoring Committee
DOLFIN: Developmental Outcomes of Long-term Feed Supplementation in Neonates
EDD: Estimated date of delivery
EPA: Eicosapentaenoic acid
EuroQoL EQ-5D-5L: EuroQol five-dimension scale questionnaire
HEAP: Health Economics Analysis Plan
HIE: Hypoxic-ischemic encephalopathy
HTA: Health Technology Assessment
ICER: Incremental Cost-Effectiveness Ratio
IGSQ: Infant Gastrointestinal Symptom Questionnaire
INFANT: Computerised interpretation of fetal heart rate during labour
INIS: International Neonatal Immunotherapy Study
IQ: Intelligence quotient
ITT: Intention-to-treat
LCPUFA: Long-chain polyunsaturated fatty acid
MHRA: Medicines and Healthcare products Regulatory Agency
N3RO: N-3 Fatty Acids for Improvement in Respiratory Outcomes randomised controlled trial
NHS: National Health Service
NIHR: National Institute for Health and Care Research
NNU: Neonatal Unit
NPEU: National Perinatal Epidemiology Unit
NuTH: Newcastle upon Tyne Hospitals NHS Foundation Trust
PAG: Parent advisory group
PARCA-R: Parent Report of Children’s Abilities-Revised
PMG: Project Management Group
PPI: Patient and public involvement
QALY: Quality-Adjusted Life Year
RCT: Randomised Controlled Trial
REC: Research Ethics Committee
RR: Risk Ratio
SAE: Serious Adverse Event
SAP: Statistical Analysis Plan
SD: Standard Deviation
SDQ: Strengths and Difficulties Questionnaire
SEN: Special Educational Needs
SIFT: Speed of Increasing Milk Feeds Trial
SOP: Standard Operating Procedures
TSC: Trial Steering Committee
UMP: Uridine-5′-monophosphate
USAR: Unexpected Serious Adverse Reaction
UK: United Kingdom
WASI-II: Wechsler Abbreviated Scale of Intelligence, Second Edition
WHO: World Health Organization

## Footnotes

1 Linsell L, Johnson S, O’Reilly H, Wolke D, Morris J, Kurinczuk J,

## References

[1] Johnson S, Hennessy E, Smith R, Trikic R, Wolke D, Marlow N. Academic attainment and special educational needs in extremely preterm children at 11 years of age: the EPICure study. Arch Dis Child Fetal Neonatal Ed. 2009;94(4):F283-9. 19282336 10.1136/adc.2008.152793

[2] Johnson S, Hennessy E, Smith R, Trikic R, Wolke D, Marlow N. Academic attainment and special educational needs in extremely preterm children at 11 years of age: the EPICure study. Arch Dis Child Fetal Neonatal Ed. 2009;94(4):F283-9. 19282336 10.1136/adc.2008.152793

[3] Johnson S, Hennessy E, Smith R, Trikic R, Wolke D, Marlow N. Academic attainment and special educational needs in extremely preterm children at 11 years of age: the EPICure study. Arch Dis Child Fetal Neonatal Ed. 2009;94(4):F283-9. 19282336 10.1136/adc.2008.152793

[4] Selman C, Rheanna M, Lee K, Anderson P, Burnett A, Garland SM, et al. Health-related quality of life in adults born extremely preterm or with extremely low birth weight in the postsurfactant era: a longitudinal cohort study. Arch Dis Child Fetal Neonatal Ed. 2023;108(6):581–5. 36997308 10.1136/archdischild-2022-325230

[5] Benestad MR, Drageset J, Vollsæter M, Hufthammer KO, Halvorsen T, Vederhus BJ. Health-related quality of life in two birth cohorts of extremely preterm born adults. Acta Paediatr. 2024;113(6):1288–97. 38353348 10.1111/apa.17146

[6] Bilgin A, Mendonca M, Wolke D. Preterm birth/low birth weight and markers reflective of wealth in adulthood: a meta-analysis. Pediatrics. 2018;142(1):e20173625. 29875181 10.1542/peds.2017-3625

[7] Lindstrom K, Lagerroos P, Gillberg C, Fernell E. Teenage outcome after being born at term with moderate neonatal encephalopathy. Pediatr Neurol. 2006;35(4):268–74. 16996401 10.1016/j.pediatrneurol.2006.05.003

[8] Marlow N, Rose AS, Rands CE, Draper ES. Neuropsychological and educational problems at school age associated with neonatal encephalopathy. Arch Dis Child Fetal Neonatal Ed. 2005;90(5):8-F387. 10.1136/adc.2004.067520 PMC1721935 16113154

[9] Perez A, Ritter S, Brotschi B, Werner H, Caflisch J, Martin E, et al. Long-term neurodevelopmental outcome with hypoxic-ischemic encephalopathy. J Pediatr. 2013;163(2):9. – 459. 23498155 10.1016/j.jpeds.2013.02.003

[10] Schreglmann M, Ground A, Vollmer B, Johnson MJ. Systematic review: long-term cognitive and behavioural outcomes of neonatal hypoxic-ischaemic encephalopathy in children without cerebral palsy Acta Paediatr. 2020;109(1):10. – 30. 31002422 10.1111/apa.14821

[11] Innis SM. Dietary omega 3 fatty acids and the developing brain. Brain Res. 2008;1237:11. – 43. 18789910 10.1016/j.brainres.2008.08.078

[12] Cansev M, Marzloff G, Sakamoto T, Ulus IH, Wurtman RJ. Giving uridine and/or docosahexaenoic acid orally to rat dams during gestation and nursing increases synaptic elements in brains of weanling pups. Dev Neurosci. 2009;31(3):12–92. 10.1159/000193394 19145070

[13] Sakamoto T, Casey M. RJ Wurtman 2007 Oral supplementation with docosahexaenoic acid and uridine-5′-monophosphate increases dendritic spine density in adult gerbil hippocampus. Brain Res. 2007;1182:13. – 9. 17950710 10.1016/j.brainres.2007.08.089 PMC2140951

[14] Georgieff MK. Nutrition and the developing brain: nutrient priorities and measurement. Am J Clin Nutr. 2007;85(2):14.-620S. 17284765 10.1093/ajcn/85.2.614S

[15] Kostovic I, Rakic P. Developmental history of the transient subplate zone in the visual and somatosensory cortex of the macaque monkey and human brain. J Comp Neurol. 1990;297:15. – 70. 2398142 10.1002/cne.902970309

[16] Brandt MJV, Nijboer C, Nessel I, Mutshiya TR, Michael-Titus AT, Counotte DS, et al. Nutritional supplementation reduces lesion size and neuroinflammation in a sex-dependent manner in a mouse model of perinatal hypoxic-ischemic brain injury. Nutrients. 2021;14(1):16. 35011052 10.3390/nu14010176 PMC8747710

[17] Zhang Z, Fulgoni V. PM Kris-Etherton SH Mitmesser 2018 Dietary intakes of EPA and DHA omega-3 fatty acids among US childbearing-age and pregnant women: an analysis of NHANES 2001–2014. Nutrients. 2018;10(4):17. 29597261 10.3390/nu10040416 PMC5946201

[18] Harris WS, Baack ML. Beyond building better brains: bridging the docosahexaenoic acid (DHA) gap of prematurity. J Perinatol. 2014;35(1):18. – 7. 25357095 10.1038/jp.2014.195 PMC4281288

[19] Hu R, Xu J, Hua Y, Li Y, Li J. Could early life DHA supplementation benefit neurodevelopment? A systematic review and meta-analysis. Front Neurol. 2024;15:19. 10.3389/fneur.2024.1295788. (PMID: 38645744; PMCID: PMC11032049). 38645744 10.3389/fneur.2024.1295788 PMC11032049

[20] Beyerlein A, Hadders-Algara M, Kennedy K, Fewtrell M, Singhal A, Rosenfeld E, et al. Infant formula supplementation with long-chain polyunsaturated fatty acids has no effect on Bayley developmental scores at 18 months of age–IPD meta-analysis of 4 large clinical trials. J Pediatr Gastroenterol Nutr. 2010;50(1):20. – 84. 19881391 10.1097/MPG.0b013e3181acae7d

[21] Gould JF, Makrides M, Gibson RA, Sullivan TR, McPhee AJ, Anderson PJ, et al. Neonatal docosahexaenoic acid in preterm infants and intelligence at 5 years. N Engl J Med. 2022;387(3):21. – 88. 36300974 10.1056/NEJMoa2206868

[22] Marc I, Piedbouef B, Lacaze-Masmonteil T, Fraser W, Mâsse B, Mohamed I, et al. Effect of maternal docosahexaenoic acid supplementation on bronchopulmonary dysplasia-free survival in breastfed preterm infants: a randomized clinical trial. JAMA. 2020;324(2):22. – 67. 32662862 10.1001/jama.2020.8896 PMC7361648

[23] Marc I, Lavoie P, Sullivan TR, Pronovost E, Boutin A, Beltempo M, Guillot M, Gould JF, Simonyan D, McPhee A, Mohamed I, Moore L, Makrides M. High-dose docosahexaenoic acid for bronchopulmonary dysplasia severity in very preterm infants: a collaborative individual participant data meta-analysis. Am J Clin Nutr. 2025;121(4):826–34. 10.1016/j.ajcnut.2025.01.004 PMC12002185 40180500

[24] Andrew MJ, Paar J, Montague-Johnson C, Laler K, Holmes J, Baker B, et al. Neurodevelopmental outcome of nutritional intervention in newborn infants at risk of neurodevelopmental impairment: the Dolphin neonatal double-blind randomized controlled trial. Dev Med Child Neurol. 2018;60(9):24. – 905. 29806081 10.1111/dmcn.13914

[25] Andrew MJ, Paar J, Montague-Johnson C, Laler K, Qi C, Baker B, et al. Nutritional intervention and neurodevelopmental outcome in infants with suspected cerebral palsy: the Dolphin infant double-blind randomized controlled trial. Dev Med Child Neurol. 2018;60(9):25. – 13. 29023666 10.1111/dmcn.13586

[26] Andrew MJ, Paar J, Montague-Johnson C, Braddick O, Laler K, Williams N, et al. Optimising nutrition to improve growth and reduce neurodisabilities in neonates at risk of neurological impairment, and children with suspected or confirmed cerebral palsy. BMC Pediatr. 2015;15:26. 25885548 10.1186/s12887-015-0339-2 PMC4389808

[27] Bayley N. The Bayley scales of infant and toddler development. 3 ed. San Antonio, TX: Pearson; 2006

[28] Kaufman AS KN. Kaufman assessment battery for children. Circle Pines: American Guidance Service; 1983

[29] Atkinson J, Braddick O, Montague-Johnson C, et al. Visual attention and dietary supplementation in children with perinatal brain injury. Dev Med Child Neurol. 2021;64(3):29. – 6. 34449080 10.1111/dmcn.15017

[30] Johnson S, Bountziouka V, Brocklehurst P, Linsell L, Marlow N, Wolke D, et al. Standardisation of the parent report of children’s abilities-revised (PARCA-R): a norm-referenced assessment of cognitive and language development at age 2 years. Lancet Child Adolesc Health. 2019;3(10):30. – 12. 31402196 10.1016/S2352-4642(19)30189-0

[31] National Institute for Health and Care Excellence. Developmental follow-up of children and young people born preterm. NICE Guidance NG72; 2017 28837304

[32] International Consortium for Health Outcomes Measurement. Preterm and Hospitalized Newborn Health Standard Set. ICHOM; 2020

[33] Goodman R. Strengths and Difficulties Questionnaire (SDQ) [Database record]. APA PsycTests. 1997. 10.1037/t00540-000

[34] Squires J, Bricker D. Ages & Stages Questionnaires®, Third Edition (ASQ®-3): A Parent-Completed Child Monitoring System. Baltimore: Paul H. Brookes Publishing Co., Inc; 2009

[35] WHO Multicentre Growth Reference Study Group. WHO child growth standards based on length/height, weight and age. Acta Paediatr Suppl. 2006;450:35. – 85. 10.1111/j.1651-2227.2006.tb02378.x. (PMID: 16817681). 16817681 10.1111/j.1651-2227.2006.tb02378.x

[36] Wright CM, Inskip HM, Godfrey K, Williams AF, Ong KK. Monitoring head size and growth using the new UK-WHO growth standard. Arch Dis Child. 2011. 10.1136/adc.2010.200030. 21285227 10.1136/adc.2010.200030 PMC3685130

[37] Freeman JV, Cole TJ, Chinn S, Jones PRM, White EM, Preece MA. Cross sectional stature and weight reference curves for the UK, 1990. Arch Dis Child. 1995;73:37–24. 10.1136/adc.73.1.17 PMC1511167 7639543

[38] Herdman M, Gudex C, Lloyd A, Janssen M, Kind P, Parkin D, et al. Development and preliminary testing of the new five-level version of EQ-5D (EQ-5D-5L). Qual Life Res. 2011;20(10):1727–36. 21479777 10.1007/s11136-011-9903-x PMC3220807

[39] Rabin R. EQ-5D-5L User Guide: Basic information on how to use the EQ-5D-5L instrument. Euroqol Group; 2011.

[40] Hout B, van MF Janssen YS Feng T Kohlmann J Busschbach DE Golicki A Lloyd L Scalone P Kind AS Pickard,. Interim scoring for the EQ-5D-5L: mapping the EQ-5D-5L to EQ-5D-3L value sets. Value Health. 2012;15:40. – 15. 22867780 10.1016/j.jval.2012.02.008

[41] Riley AWTJ, Yao M, Bevans KB, DeRusso PA. Validation of a parent report questionnaire: the infant gastrointestinal symptom questionnaire. Clin Pediatr (Phila). 2015;54(12):41–74. 10.1177/0009922815574075 PMC4564761 25758425

[42] Field DSE, Davies T, Manktelow B, Johnson S, Boyle E, Draper ES. Evaluation of the use of a parent questionnaire to provide later health status data: the PANDA study. Arch Dis Child Fetal Neonatal Ed. 2016;101(4):42. – 8. 26463120 10.1136/archdischild-2015-309247

[43] Dorling J, Abbott J, Berrington J, Bosiak B, Bowler U, Boyle E, et al. Controlled trial of two incremental milk-feeding rates in preterm infants. N Engl J Med. 2019;381(15):43. – 43. 31597020 10.1056/NEJMoa1816654

[44] Horta BL, dMC Loret CG Victora,. Breastfeeding and intelligence: a systematic review and meta-analysis. Acta Paediatr. 2015;104(467):44. – 9. 26211556 10.1111/apa.13139

[45] Jones KC, Weatherly H, Birch S, Castelli A, Chalkley M, Dargan A, et al. Unit Costs of Health and Social Care 2023 Manual. Kent (UK): Personal Social Services Research Unit; 2023.

[46] Janson E, Koolschijn P, Schipper L, Boerma TD, Wijnen FNK, de Boode WP, et al. Dolphin CONTINUE: a multi-center randomized controlled trial to assess the effect of a nutritional intervention on brain development and long-term outcome in infants born before 30 weeks of gestation. BMC Pediatr. 2024;24(1):46. 38849784 10.1186/s12887-024-04849-1 PMC11157897

